# Construction of Fire Safe Thermoplastic Polyurethane/Reduced Graphene Oxide Hierarchical Composites with Electromagnetic Interference Shielding

**DOI:** 10.3390/molecules29133108

**Published:** 2024-06-29

**Authors:** Yan Liu, Ansheng Yao, Libi Fu, Shiwei Xie, Yijie Zhang, Peihui Xu, Yuezhan Feng, Yongqian Shi

**Affiliations:** 1 College of Environment and Safety Engineering, Fuzhou University, 2 Xueyuan Road, Fuzhou 350116, China; liu18050901@163.com (Y.L.); yaoansheng@163.com (A.Y.); xsw106766@163.com (S.X.); zyj12132000@163.com (Y.Z.); phxu@fuz.edu.cn (P.X.); 2 College of Civil Engineering, Fuzhou University, 2 Xueyuan Road, Fuzhou 350116, China; 3 Key Laboratory of Materials Processing and Mold, Ministry of Education, National Engineering Research Center for Advanced Polymer Processing Technology, Zhengzhou University, Zhengzhou 450002, China; yzfeng@zzu.edu.cn

**Keywords:** thermoplastic polyurethane, self-assembly, flame retardancy, electromagnetic shielding

## Abstract

Incorporating outstanding flame retardancy and electromagnetic interference shielding effectiveness (EMI SE) into polymers is a pressing requirement for practical utilization. In this study, we first employed the principles of microencapsulation and electrostatic interaction-driven self-assembly to encapsulate polyethyleneimine (PEI) molecules and Ti_3_C_2_T_x_ nanosheets on the surface of ammonium polyphosphate (APP), forming a double-layer-encapsulated structure of ammonium polyphosphate (APP@PEI@Ti_3_C_2_T_x_). Subsequently, flame-retardant thermoplastic polyurethane (TPU) composites were fabricated by melting the flame-retardant agent with TPU. Afterwards, by using air-assisted thermocompression technology, we combined a reduced graphene oxide (rGO) film with flame-retardant TPU composites to fabricate hierarchical TPU/APP@PEI@Ti_3_C_2_T_x_/rGO composites. We systematically studied the combustion behavior, flame retardancy, and smoke-suppression performance of these composite materials, as well as the flame-retardant mechanism of the expansion system. The results indicated a significant improvement in the interface interaction between APP@PEI@Ti_3_C_2_T_x_ and the TPU matrix. Compared to pure TPU, the TPU/10APP@PEI@1TC composite exhibited reductions of 84.1%, 43.2%, 62.4%, and 85.2% in peak heat release rate, total heat release, total smoke release, and total carbon dioxide yield, respectively. The averaged EMI SE of hierarchical TPU/5APP@PEI@1TC/rGO also reached 15.53 dB in the X-band.

## 1. Introduction

Thermoplastic polyurethane (TPU) boasts numerous outstanding properties, including corrosion resistance, light weight, high elasticity, and robust chemical stability. It finds extensive applications in construction, medicine, transportation, military, and other fields. However, TPU is inherently flammable and releases significant heat and toxic gases during combustion, limiting its applications across various fields [1,2,3]. Furthermore, with the development of communication technology and electronics, electromagnetic radiation in our daily lives and work environments is becoming increasingly serious, posing risks to both human health and the proper functioning of devices. Unfortunately, due to the essential properties of its insulator, TPU has no role in blocking, absorbing, and reflecting electromagnetic waves [4,5,6]. Therefore, advancing the development of TPU composites with outstanding flame-retardant and electromagnetic shielding capabilities is imperative to elevate their versatility across a spectrum of applications.

Phosphorus-based flame retardants are rational polymer flame retardants due to their low smoke, low toxicity, low usage, high flame-retardant efficiency, and wide applicability [7]. With its high phosphorus content, ammonium polyphosphate (APP) is an intumescent flame retardant widely added in polymer matrices to catalyze carbonization and quench free radicals [8]. However, weak interface bonding between APP and polymers can lead to the unstable adhesion performance of APP on the polymer, reducing the material’s overall performance. It is generally believed that the interface interaction between APP and TPU can be effectively enhanced by surface modification of APP, such as microencapsulation, surface-modified technique, and self-assembly technology to enhance the refractoriness of the polymer [9,10,11]. For instance, Huang et al. [12] prepared a flame-retardant APP@LDH by electrostatic interaction, and the addition of LDH enhanced the interfacial interaction between APP and the TPU matrix. Under the same condition of 1.0 wt% LDH, the tensile strength of the TPU with 7.0 wt% APP@LDH was 32.5% higher than that of the TPU with 7.0 wt% APP/LDH, and compared with the pure TPU, the peak heat release rate (PHRR) and total heat release (THR) decreased by 74.3% and 32.2%, respectively.

Introducing conductive fillers into polymers can enhance their conductivity, improving their electromagnetic interference shielding effectiveness (EMI SE) [13]. For example, carbon black [14], reduced graphene oxide [15], and silver nanowires [16] have been used to improve the conductivity of polymer materials. Among them, reduced graphene oxide (rGO) has attracted more and more attention due to its high electrical conductivity, environmental friendliness, superior mechanical performance, and ultra-high specific surface area [17,18,19,20,21,22]. It is an ideal raw material for the attenuation of electromagnetic interference. Zahid et al. [23] prepared TPU composites incorporating reduced graphene oxide (rGO), achieving a maximum shielding efficiency of 53 dB with 2.5 wt% rGO. However, the disadvantages of the high fire risk and the high electromagnetic interference coefficient of polymers still need to be overcome.

In recent years, titanium carbide (Ti_3_C_2_T_x_, MXene) has gained widespread utilization in biomedical research, electromagnetic shielding, and energy storage, owing to its exceptional metal conductivity, impressive mechanical properties, and remarkable hydrophilicity, making it an ideal polymer nanofiller [24,25]. For example, Shi et al. [26] prepared a high-efficiency Ti_3_C_2_T_x_/Nano-Cu composite smoke suppressant by interfacial bonding method and prepared TPU/Ti_3_C_2_T_x_/Nano-Cu composites by melt-blending with TPU. The Ti_3_C_2_T_x_/Nano-Cu hybrid was uniformly distributed, and the peak smoke production rate (PSPR), peak carbon monoxide production rate (PCOPR), and total carbon monoxide yield (COTY) of the TPU nanocomposites containing 2.0 wt% Ti_3_C_2_T_x_/Nano-Cu were reduced by 66.7%, 51.8%, and 52.9%, respectively. Luo et al. [27] fabricated TPU/PCS-Mxene composites by incorporating phosphochitosan (PCS) functionalized Mxene, where with just 3 wt% of PCS-Mxene, the composites exhibited 66.7% reduction in PHRR, 21.0% reduction in THR, and 27.7% reduction in total smoke yield (TSY), compared to the pristine TPU. Wang et al. [28] prepared a composite aerogel by blending Ti_3_C_2_T_x_ and carboxymethyl cellulose nanofibers during the freeze-drying stage and then encapsulated it with TPU containing a flame retardant. The resulting composite aerogel achieved an EMI SE value of 93.5 dB in the X-band. 

In this study, we utilized the charge differences in APP, polyethyleneimine (PEI), and Ti_3_C_2_T_X_ nanosheets in an aqueous medium to prepare an APP@PEI@Ti_3_C_2_T_x_ flame retardant using an electrostatic self-assembly technique [29,30,31]. Subsequently, a multilayer TPU composite was fabricated, with TPU/APP@PEI@Ti_3_C_2_T_X_ forming the surface layer and a reduced graphene oxide (rGO) film serving as the intermediary layer, utilizing a combination of melt-blending and air-assisted thermocompression technology. The thermal stability, smoke-suppression, and flame-retardant performances of the TPU/APP@PEI@Ti_3_C_2_T_x_ composites were investigated, alongside an exploration of the impact of the rGO film on the electromagnetic shielding capabilities of the TPU material. 

## 2. Results and Discussion

### 2.1. Microstructure and Thermal Stability of APP@PEI@Ti_3_C_2_T_x_

**Figure 1:**
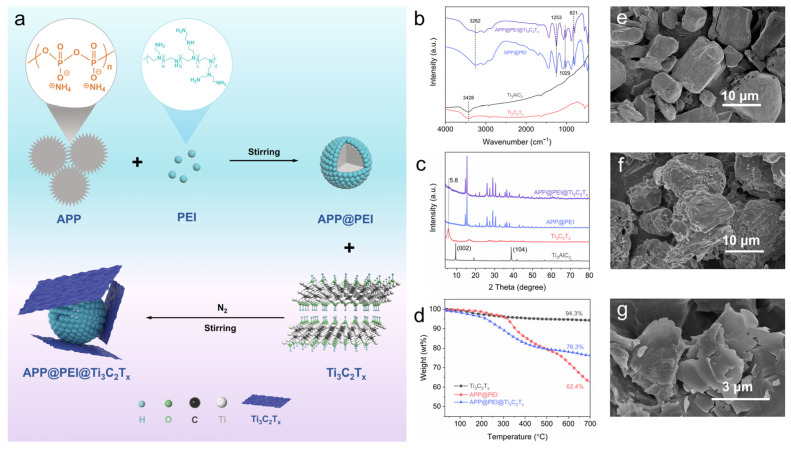
(**a**) Schematic diagrams for preparation of APP@PEI@Ti_3_C_2_T_x_; (**b**) XRD patterns and (**c**) FTIR spectra of the flame retardants; (**d**) TG curves of flame retardants; SEM images of (**e**) APP, (**f**) APP@PEI, and (**g**) APP@PEI@Ti_3_C_2_T_x_.

The synthesis diagram of flame retardant is displayed in Figure 1a. XRD patterns and FTIR spectra are used to study flame retardants’ crystal and chemical structure. As shown in Figure 1b, the (0 0 2) characteristic peak of Ti_3_C_2_T_x_ decreased to 5.8°, compared with that of Ti_3_AlC_2_, manifesting that the interlayer space of Ti_3_C_2_T_x_ nanosheets increased significantly. In addition, the complete disappearance of the (1 0 4) characteristic peak of Ti_3_C_2_T_x_ at 38.8° indicates the total removal of aluminum from Ti_3_AlC_2_. All the observations mentioned above indicate the completion of the etching process of Ti_3_AlC_2_. At the same time, the characteristic peaks of Ti_3_C_2_T_x_ and APP@PEI appeared in the XRD spectrum of APP@PEI@Ti_3_C_2_T_X_, indicating the successful synthesis of APP@PEI@Ti_3_C_2_T_x_. The FTIR test results are shown in Figure 1c. In the FTIR spectrum of Ti_3_C_2_T_x_, a characteristic absorption peak formed by the stretching vibration of –OH appears at 3428 cm^−1^. After Ti_3_C_2_T_x_ encapsulates APP@PEI, APP@PEI@Ti_3_C_2_T_x_ shows characteristic absorption peaks at 821 cm^−1^ (asymmetric stretching vibration of P–O), 1029 cm^−1^ (symmetric stretching vibration of PO_2_ and PO_3_), 1253 cm^−1^ (stretching vibration of P–O), and 3262 cm^−1^ (stretching vibration of –OH), which further confirms that the APP@PEI@Ti_3_C_2_T_x_ hybrid is successfully prepared.

As shown in Figure 1e–g, the morphologies of APP, APP@PEI, and APP@PEI@Ti_3_C_2_T_x_ were observed by SEM. Compared with the smooth surface of APP particles in Figure 1e, Figure 1f shows the irregular surface and spotty protrusions of APP particles after PEI modification, which provides good adhesion conditions for Ti_3_C_2_T_x_ nanosheets. Figure 1g shows that the surface of solid particles is wrapped by Ti_3_C_2_T_x_ sheets, further revealing that the APP@PEI@Ti_3_C_2_T_x_ hybrid is successfully synthesized.

TGA was used to study the thermal stability of flame retardant. The test results are shown in Figure 1d and Table S1. *T*_-5_, *T*_-50_, and *T*_-max_ represent the temperature corresponding to the material mass loss rate of 5%, 50%, and the maximum mass loss rate, respectively. Ti_3_C_2_T_X_ shows good thermal stability and slight degradation in the range of room temperature to 700 °C. In addition, with the increase in temperature, the mass of APP@PEI decreased significantly, mainly due to the thermal degradation of ؘ–OH and –NH_2_. Compared with APP@PEI, APP@PEI@Ti_3_C_2_T_X_ displays increased residual carbon content at 700 °C, and the improvement in thermal stability of the latter is mainly due to the introduction of Ti_3_C_2_T_x_.

### 2.2. Flame-Retardant Evaluation of TPU/APP@PEI@Ti_3_C_2_T_x_ Composites

The thermal degradation characteristics of TPU and its composites were investigated using an automated sampling synchronous thermal analyzer. The relevant results are demonstrated in Figure S2 and Table S2. Pure TPU initially degrades at 327.0 °C and has two thermal degradation stages, which is consistent with early reports [32,33]. However, TPU composites show three degradation process stages. The third stage ascribes the final carbon residue catalyzed by the flame retardant. In addition, the carbon residue of pure TPU at 700 °C is only 9.6 wt%. The *T*_-5_, *T*_-50_, and *T*_-max_ of TPU composites are lower than those of pure TPU, mainly due to the early degradation and catalysis of APP and APP@PEI. As the proportion of Ti_3_C_2_T_x_ in the flame-retardant blend increased, the thermal stability of the composites showed an increasing trend, which also reflected that Ti_3_C_2_T_x_ contributed positively to the thermal stability of the composites. In addition, the TPU composites have higher carbon residues than the pure samples. For example, the carbon residue of TPU/20APP@PEI@1TC at 700 °C is 30.1 wt%, much higher than that of a pure sample. Introducing a microencapsulated flame retardant markedly boosts the carbon residue of TPU composites, which is attributed to the physical barrier effect and superior catalytic carbonization ability of the APP@PEI@Ti_3_C_2_T_x_ hybrid. The catalytic carbonization function of APP@PEI@Ti_3_C_2_T_x_ can be observed from the TGA results, which are conducive to improving the thermal stability of TPU composites.

**Figure 2:**
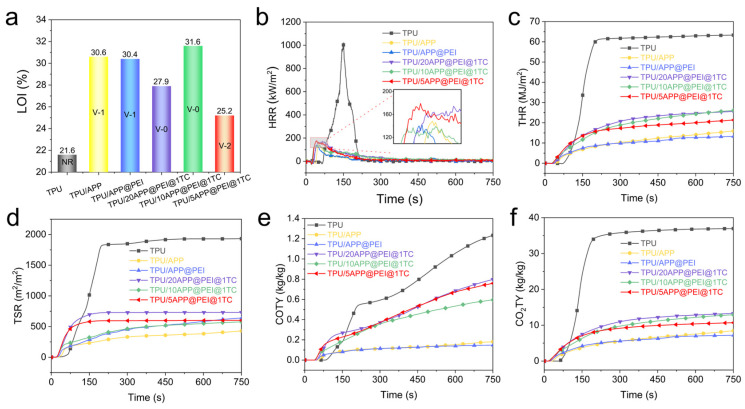
(**a**) LOI and UL-94 testing results, (**b**) HRR, (**c**) THR, (**d**) TSR, (**e**) COTY, and (**f**) CO_2_TY of TPU and its composites.

The limiting oxygen index (LOI) is the concentration of oxygen as a volume fraction of a polymer in a mixture of oxygen and nitrogen when it is just able to support its combustion [33], and the vertical combustion test is a small-scale fire test used to evaluate the flammability of polymer materials. These methods are widely employed for assessing the fire resistance properties of materials, particularly for testing the effectiveness of flame-retardant formulations in polymer materials. The relevant results of the LOI and UL-94 level tests of TPU and its composites are shown in Figure 2a. The LOI value of pure TPU is only 21.6%, and its UL-94 grade is no grade (NR), while the LOI values of the TPU composite added with 13.6 wt% APP and 13.6 wt% APP@PEI are 30.6% and 30.4%, respectively, whereas its vertical combustion test grade also only reaches UL-94 V-1 grade. It is worth noting that the LOI value of the TPU composites is significantly higher than that of pure TPU (21.6%) with the introduction of the flame retardant. For TPU composites with APP@PEI@Ti_3_C_2_T_x_, the LOI value shows irregular changes, in which the LOI value of TPU/10APP@PEI@1TC is as high as 31.6%, but the LOI values of the other two samples are only 27.9% (TPU/20APP@PEI@1TC) and 25.2% (TPU/10APP@PEI@1TC), and the vertical combustion test grade of the latter is only UL-94 V-2. As for the vertical combustion test results, only the TPU/20APP@PEI@1TC and TPU/10APP@PEI@1TC groups of samples reach the UL-94 V-0 level. The results indicate that adding an appropriate amount of APP@PEI@1TC can significantly enhance the fire-resistance performance of TPU composite materials.

A cone calorimeter (CCT) is an instrument for assessing composite materials’ flammability and fire risk [34,35]. Figure 2 and Table 1 show the CCT results for all samples. The ignition to time (TTI) of the TPU composite is lower than that of pure TPU, mainly due to the catalytic effect of intumescent flame-retardant APP and Ti_3_C_2_T_x_ nanosheets. TGA test results are also consistent with this phenomenon (Figure S2). The results show that the PHRR and THR of pure TPU were as high as 872.3 kW/m^2^ and 63.7 MJ/m^2^, respectively. After introducing 13.6 wt% APP, the PHRR, and the THR of TPU, the material decreased to 148.4 kW/m^2^ and 20.7 MJ/m^2^, respectively. However, after the introduction of hybrid flame retardant, the PHRR of the TPU/10APP@PEI@1TC composite further decreases by 84.1%, compared with that of pure TPU, indicating that Ti_3_C_2_T_x_ and APP@PEI have a good synergistic effect in decreasing the thermal release of composites.

In the case of fire, smoke is the chief culprit that hinders people from escaping the fire [36,37]. As shown in Table 1 and Figure 2, the PSPR and total smoke release (TSR) of pure TPU reach 0.1868 m^2^/s and 2058.8 m^2^/m^2^, respectively. However, by adding APP and microencapsulated APP additives, the smoke emission of composites is effectively controlled. For instance, the PSPR and TSR of TPU/APP are decreased by 50.3% and 74.3%, respectively, compared to pure TPU. However, with the addition of APP and APP@PEI, the smoke emission of the TPU composites is not further reduced, and the smoke-suppression performance is not as good as that of the former. Figure 2e,f and Table 1 show all samples’ CO and CO_2_ emissions during combustion. The PCOPR values of TPU/APP and TPU/APP@PEI are 0.0031 g/s and 0.0028 g/s, respectively, 53.0% and 57.6% lower than pure TPU. In addition, the COTY of all samples shows the same trend as their PCOPR. For instance, the COTY of TPU/APP is 0.2315 kg/kg, which is 80.8% less than that of pure TPU (1.2117 kg/kg). However, after the incorporation of APP@PEI, APP@PEI@Ti_3_C_2_T_x_ hybrid flame retardant, the PCOPR, COTY, peak carbon dioxide production rate (PCO_2_PR), and CO_2_TY of the composite are increased, compared with those of TPU/APP and TPU/APP@PEI. The above results indicate that the combination of Ti_3_C_2_T_x_ and APP@PEI is not conducive to suppressing the release of toxic gases and the generation of smoke during combustion.

### 2.3. Flame-Retardant Mechanism

By analyzing the morphology and structure of carbon residues after CCT, the flame-retardant mechanism of TPU/APP@PEI@Ti_3_C_2_T_x_ composite materials can be studied. The digital images of the carbon residues for all samples are depicted in Figure S3a–e. The carbon residue of pure TPU is sparse, porous, and accompanied by cracks (Figure S3a), showing a fragile and loose structure. After adding 13.6 wt% of APP@PEI and APP@PEI@Ti_3_C_2_T_x_, the carbon slag of the TPU composites becomes continuous and tight (Figure S3b,c). After introducing APP@PEI@Ti_3_C_2_T_x_, the TPU/APP@PEI@Ti_3_C_2_T_x_ composite shows a more constant and dense carbon layer (Figure S3d,e). Furthermore, the compactness and completeness of the carbon residue also increase with the increase in the amount of Ti_3_C_2_T_x_. Adding intumescent flame-retardant APP substantially boosts the carbon slag content, protecting the internal composites and significantly enhancing their flame retardancy. These results show that adding Ti_3_C_2_T_x_ nanosheets effectively improves the quality of the carbon layer, and the denser carbon layer effectively suppresses the smoke generation and toxic gas release of TPU during combustion, greatly reducing the fire risk of the composite.

**Figure 3:**
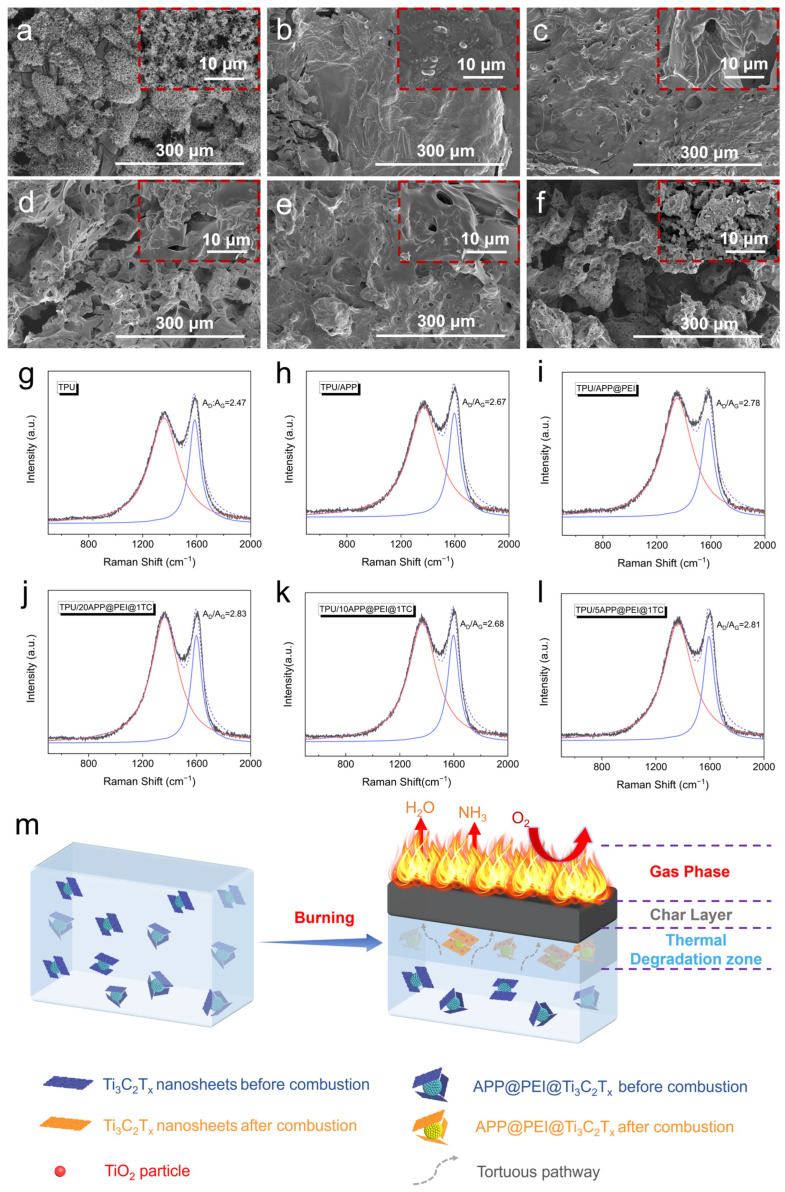
(**a**–**f**) SEM images of the char residues after the CCT test; (**g**–**l**) Raman spectra of carbon residues of TPU and its composites; (**m**) schematic representation for the flame-retardant mechanism of TPU/APP@PEI@Ti_3_C_2_T_x_ composite.

SEM and Raman tests were conducted to elucidate the flame-retardant mechanism and investigate the carbon slag of all samples after the CCT test (Figure 3). Pure TPU exhibits a loose structure with numerous holes in its carbon slag (Figure 3a). In contrast, the carbon slag of TPU/APP becomes denser and more continuous with fewer holes (Figure 3b). TPU/APP@PEI displays a reduced number of smaller holes compared to the former (Figure 3c). However, adding APP@PEI@Ti_3_C_2_T_x_ results in a more fractured carbon slag with many holes, explaining its altered flame-retardant performance at the micro level. (Figure 3d,f).

In the Raman spectra of the carbon slag of TPU composites (Figure 3h–l), two distinct peaks at around 1385 cm^−1^ and 1586 cm^−1^ correspond to the D band and G band, respectively [38]. The area ratio of the D band to the G band (*A*_D_/*A*_G_) is usually used to represent the graphitization degree of carbon residue. It is widely acknowledged that a lower *A*_D_/*A*_G_ value indicates a higher level of graphitization in the residue, leading to enhanced flame retardancy and smoke-suppression properties in polymer materials [39,40,41]. The *A*_D_/*A*_G_ ratio of the TPU/APP carbon slag is determined to be 2.67, surpassing the value of pure TPU (2.47). The *A*_D_/*A*_G_ values of the carbon residue in the TPU/APP@PEI, TPU/20APP@PEI@1TC, TPU/10APP@PEI@1TC, and TPU/5APP@PEI@1TC samples are higher than 2.67. These phenomena are not consistent with the CCT results and SEM of char residues, which do not imply that the char slag has become loose but rather that the addition of the swelling flame retardant has caused the char slag to become more expanded with more dispersed residual char points [42]. Additional XRD and FTIR tests were carried out on the carbon slag of TPU and its composites to explore the flame-retardant mechanism of composites further, as shown in Figure S4a,b. The XRD pattern of TPU/APP@PEI@Ti_3_C_2_T_x_ (Figure S4a) reveals prominent peaks at 25.2° and 27.7°, corresponding to the characteristic peaks of TiO_2_. This indicates that Ti_3_C_2_T_x_ undergoes oxidation to form TiO_2_ during aerobic combustion. The presence of TiO_2_ significantly enhances the quality of the carbon layer, thereby improving the flame retardancy of the composite. Moreover, Figure S4b shows that the characteristic peak of carbon slag in all TPU composites is consistent with that of pure TPU. It can be seen that the addition of flame-retardant filler will not change the combustion path of the TPU material.

Drawing from the preceding analysis, we can outline the flame-retardant mechanism of TPU/APP@PEI@Ti_3_C_2_T_x_ composites (Figure 3m). On the one hand, APP will decompose when heated to produce a large amount of water vapor and ammonia, and making many non-combustible gases will greatly reduce the oxygen concentration around. On the other hand, APP and Ti_3_C_2_T_x_ exhibit exceptional catalytic carbonization capabilities, forming a compact solid carbon layer upon thermal degradation. This layer effectively hinders heat conduction and the infiltration of volatile substances. Furthermore, the labyrinth effect of Ti_3_C_2_T_x_ nanosheets hinders the emission of pyrolysis gases during thermal decomposition. Therefore, the introduction of APP@PEI@Ti_3_C_2_T_x_ hybrid flame retardant can significantly reduce the fire hazard of TPU composites.

### 2.4. EMI Shielding Assessment of Hierarchical TPU/APP@PEI@Ti_3_C_2_T_x_/rGO Composites

The anti-electromagnetic interference ability of pure TPU is almost zero, which will seriously affect equipment safety and people’s health. We used a vector network analyzer to measure the EMI SE value of all samples at the X-band. To investigate the electromagnetic shielding mechanism of hierarchical TPU/APP@PEI@Ti_3_C_2_T_x_/rGO composites featuring a multilayer structure. EMI SE is composed of three parts: absorption (*SE*_A_), reflection (*SE*_R_), and multiple reflections (SEMRs). Considering that the thickness of the composite is less than the depth of the skin, the last one can be ignored here [43].

Furthermore, the *SE*_T_, *SE*_A_, and *SE*_R_ of all TPU samples and rGO film are calculated according to the following equations:

(1) $${\mathrm{SE}}_{R}=-10\;\log\left(1-{\left|S_{11}\right|}^{2}\right)$$

(2) $${{\mathrm{SE}}_{A}}_{\;}=10\;\log\left(T⁄1-R\right)=10\;\log\left({\left|S_{21}\right|}^{2}/(1-{\left|S_{11}\right|}^{2})\right)$$

*SE*_T_ = *SE*_R_ + *SE*_A_ (3)

**Figure 4:**
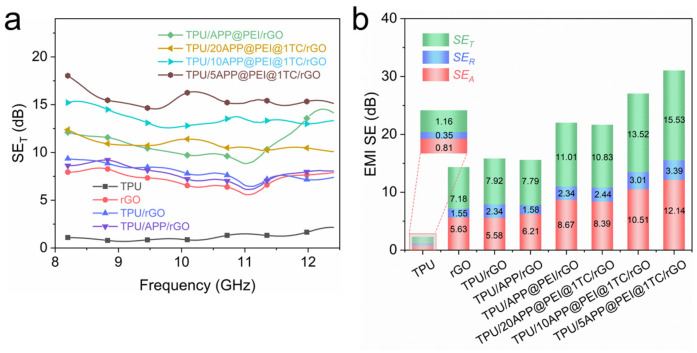
(**a**) Total EMI SE curves, (**b**) the averaged *SE*_T_, *SE*_R,_ and *SE*_A_ values.

The *SE*_T_, *SE*_A_, and *SE*_R_ calculation results of all TPU samples and the rGO film are shown in Figure 4. Figure 4a shows the total shielding efficiency (*SE*_T_) as a function of the X-band (8.2–12.4 GHz) frequency of the multilayer hierarchical TPU/APP@PEI@Ti_3_C_2_T_x_/rGO composite. It can be seen that the *SE*_T_ value of pure TPU is close to zero, while the *SE*_T_ value of the rGO film is 7.18 dB. Compared with pure TPU and rGO films, the SET value of multilayer TPU/rGO composites is 7.92 dB, slightly exceeding the rGO film. This suggests that the presence of the rGO film causes TPU composites with a multilayer structure to absorb a portion of the electromagnetic waves that penetrate through its interior. Moreover, the higher SET value of TPU/APP@PEI, compared to TPU/APP, is primarily due to the conductive network established by APP and PEI through electrostatic interaction-driven self-assembly. This network further enhances the SET of the multilayer TPU composite structure [44]. Notable is the increase in the SET value of hierarchical TPU/APP@PEI@Ti_3_C_2_T_x_/rGO composites with a multilayer structure upon the addition of Ti_3_C_2_T_x_. Additionally, the SET value of TPU composites rises in tandem with the increase in Ti_3_C_2_T_x_ loading. This is attributed to the conductive network formed by Ti_3_C_2_T_x_ nanosheets in the composite and the large hydrogen bonding with APP and PEI, which will also enhance the electromagnetic shielding efficiency of the composite. Moreover, it is easy to observe from Figure 4b that there are dominant *SE*_A_ and subordinate *SE*_R_ in the *SE*_T_ value of all samples, which means that the absorption effect plays a key role in attenuating electromagnetic waves. The above results demonstrate that hierarchical TPU/APP@PEI@Ti_3_C_2_T_x_/rGO composite with multilayer structure has good electromagnetic shielding performance.

## 3. Materials and Methods

### 3.1. Materials and Methods

The Supplementary Materials provide raw materials and testing methods; GO was synthesized via Hummers’ method [45].

### 3.2. Preparation of Ti_3_C_2_T_x_ Nanosheets 

According to the improved HF etching method, Ti_3_C_2_T_x_ nanosheets were prepared from Ti_3_AlC_2_ [46,47]. Firstly, 1.00 g of Ti_3_AlC_2_ and 1.56 g of LiF were slowly introduced into a centrifuge tube containing 20 mL of HCl under magnetic stirring and maintained at 35 °C for 2 d. Subsequently, the dispersion was washed with deionized water until neutral. Finally, the Ti_3_C_2_T_x_ dispersion was stirred in an ice bath for 0.5 h and then centrifuged at 8500 rpm for 5 min to obtain Ti_3_C_2_T_x_ nanosheets.

### 3.3. Preparation of rGO Film

Initially, the reduction of the GO aqueous solution was carried out using a chemical reduction method. Subsequently, a specified quantity of N_2_H_4_·H_2_O was added to the GO aqueous solution, followed by magnetic stirring for 6 h to obtain an rGO suspension. Subsequently, 100.00 g of the rGO suspension was transferred to a Büchner funnel containing filter paper (90 mm in diameter, pore size of 0.2 μm) through vacuum filtration, forming an rGO film upon drying.

### 3.4. Synthesis of APP@PEI@Ti_3_C_2_T_x_

First, 20.00 g of APP was dispersed in 400 mL of deionized water and mechanically stirred for 0.5 h to form a uniform dispersion. Then 2.00 g of PEI was dissolved in deionized water, magnetically stirred for 0.5 h, then slowly added to the above dispersion, and mechanically stirred at room temperature for 2 h. The solvent was removed by centrifugation, the solid was washed with deionized water and absolute ethanol, and then the solid was dried at 80 °C for 24 h to obtain the intermediate product APP@PEI. A certain amount of Ti_3_C_2_T_x_ dispersion was added to a 500 mL three-necked flask, and the suspension was formed by ultrasonic stirring for 0.5 h under ice bath conditions. Then, a certain amount of APP@PEI dispersed in deionized water was mechanically stirred for 0.5 h, and the Ti_3_C_2_T_x_ dispersion was added dropwise to the above solution under nitrogen conditions (APP@PEI and Ti_3_C_2_T_x_ solutions with solute mass ratios of 20:1, 10:1, and 5:1, respectively). After mechanical stirring for 4 h, the solid obtained by suction filtration and washing is dried in a vacuum at 80 °C for 24 h to obtain APP@PEI@Ti_3_C_2_T_x_.

### 3.5. Fabrication of TPU/APP@PEI@Ti_3_C_2_T_x_ Composites

TPU composites were prepared by introducing APP@PEI@Ti_3_C_2_T_x_ into a TPU via melt-blending methods and air-assisted thermocompression technology. The flame-retardant additive and TPU were melt-blended using a twin roller at 185 °C, followed by hot-pressing the resulting product at 180 °C to obtain standard samples for characterization. Table 2 shows the formulation of TPU composites where, for TPU/xAPP@PEI@yTC composites, x/y is APP@PEI and the mass ratio of Ti_3_C_2_T_x_, while TC represents Ti_3_C_2_T_x_. It can be observed from Figure S1 that these TPU composites exhibit good compatibility between flame retardants and the TPU host.

### 3.6. Construction of Hierarchical TPU/APP@PEI@Ti_3_C_2_T_x_/rGO

TPU and its composites with a thickness of 0.5 mm were prepared by combining melt-blending with hot-pressing. Hierarchical TPU composites with inserted rGO films were prepared as follows. Typically, a layer of 0.5 mm TPU (or its composites) was hot-pressed with a piece of rGO film. Subsequently, after preheating, another 0.5 mm TPU sheet without an rGO film was hot-pressed together with the previous sample. The hierarchical films with a thickness of approximately 1.0 mm were labeled as hierarchical TPU/APP@PEI@Ti_3_C_2_T_x_/rGO.

## 4. Conclusions

In summary, a hierarchical TPU/APP@PEI@Ti_3_C_2_T_x_/rGO, an excellent flame-retardant composite material with EMI-shielding properties, was successfully fabricated by using melt-blending and novel air-assisted thermocompression technology. Relevant test results demonstrated that the dispersion of APP@PEI@Ti_3_C_2_T_x_ in the TPU matrix was excellent, and it exhibited robust interfacial interactions with the TPU matrix. In addition, the thermal stability of the TPU material exhibited a remarkable enhancement following the incorporation of the APP@PEI@Ti_3_C_2_T_x_ hybrid. The CCT results indicate that the incorporation of 13.6% APP@PEI@Ti_3_C_2_T_x_ led to a significant reduction of 84.1% in PHRR, 43.7% in PSPR, 62.4% in TSR, and 85.2% in PCO_2_PR for TPU/10APP@PEI@1TC composites. Furthermore, the introduction of APP@PEI@Ti_3_C_2_T_x_ substantially enhanced the electromagnetic shielding efficiency of the composites. This study provides a novel and effective method for preparing composites with multiple functionalities, which are expected to be used in flame-retardant and anti-interference applications.

## Figures and Tables

**Table 1:** CCT results of TPU and its composites under a heat reflux of 35 kW/m^2^.

Sample No.	TTI /s	PHRR /kWm^−2^	THR /MJ·m^−2^	PSPR /m^2^·s^−1^	TSR /m^2^·m^−2^	PCOPR /g·s^−1^	COTY /kg·kg^−1^	PCO_2_PR /g·s^−1^	CO_2_TY /kg·kg^−1^
TPU	65	872.3	63.7	0.1868	2058.8	0.0066	1.2117	0.4803	37.9
TPU/APP	38	148.4	20.7	0.0928	529.8	0.0031	0.2315	0.0757	10.0
TPU/APP@PEI	28	142.7	14.8	0.0966	689.6	0.0028	0.1582	0.0689	7.5
TPU/20APP@PEI@1TC	31	174.4	29.1	0.1125	730.7	0.0048	1.0359	0.0878	14.6
TPU/10APP@PEI@1TC	22	138.9	36.2	0.1051	774.2	0.0041	0.8564	0.0712	15.6
TPU/5APP@PEI@1TC	23	179.1	25.6	0.1267	603.4	0.0053	0.9102	0.0797	11.4

**Table 2:** Specific formulation table of TPU composite materials.

Sample Number	TPU /g	APP /g	APP@PEI /g	APP@PEI@Ti_3_C_2_T_x_ /g	APP@PEI/Ti_3_C_2_T_x_ /%·%^−1^
TPU	60.0	0.0	0.0	0.0	/
TPU/APP	52.8	7.2	0.0	0.0	/
TPU/APP@PEI	52.8	0.0	7.2	0.0	/
TPU/20APP@PEI@1TC	52.8	0.0	0.0	7.2	20/1
TPU/10APP@PEI@1TC	52.8	0.0	0.0	7.2	10/1
TPU/5APP@PEI@1TC	52.8	0.0	0.0	7.2	5/1

## Data Availability

Data are contained within the article and Supplementary Materials.

## Supplementary Materials

The following supporting information can be downloaded at: https://www.mdpi.com/article/10.3390/molecules29133108/s1, Materials and Methods; Figure S1: SEM images of the cross-sections of TPU and its composite materials: (a) TPU, (b) TPU/APP, (c) TPU/APP@PEI, (d) TPU/20APP@PEI@1TC, (e) TPU/10APP@PEI@1TC, and (f) TPU/5APP@PEI@1TC; Figure S2: (a) TG and (b) DTG curves of TPU and its composites; Figure S3: Digital photographs of the char residues for TPU and its composite materials after combustion test: (a) TPU, (b) TPU/APP, (c) TPU/APP@PEI, (d) TPU/20APP@PEI@1TC, (e) TPU/10APP@PEI@1TC, and (f) TPU/5APP@PEI@1TC; Figure S4: (a) XRD patterns and (b) FTIR spectra of char residues for TPU and its nanocomposites; Table S1: TGA test data of flame retardants under nitrogen conditions; Table S2: TG results of TPU and its composites under nitrogen condition.
