# Supplementary material for: Construction of Fire Safe Thermoplastic Polyurethane/Reduced Graphene Oxide Hierarchical Composites with Electromagnetic Interference Shielding
Source: Molecules. 2024 Jun 29;29(13):3108. doi: 10.3390/molecules29133108 (PMC11243064; doi:10.3390/molecules29133108)
Supplement: Supplementary file 1 [file molecules-29-03108-s001.zip › molecules-3031145-supplementary.pdf]

Supplementary Material for

Construction of Fire Safe Thermoplastic Polyurethane/Reduced Graphene Oxide Hierarchical Composites with Electromagnetic Interference Shielding

Yan Liu [1,†], Ansheng Yao [1,†], Libi Fu [2,*], Shiwei Xie [1], Yijie Zhang [1], Peihui Xu [1], Yuezhan Feng [3] and Yongqian Shi [1,*]

[1] College of Environment and Safety Engineering, Fuzhou University, 2 Xueyuan Road, Fuzhou 350116, China; liu18050901@163.com (Y.L.); yaoansheng@163.com (A.Y.); xsw106766@163.com (S.X.); zyj12132000@163.com (Y.Z.); phxu@fuz.edu.cn (P.X.)
[2] College of Civil Engineering, Fuzhou University, 2 Xueyuan Road, Fuzhou 350116, China
[3] Key Laboratory of Materials Processing and Mold, Ministry of Education, National Engineering Research Center for Advanced Polymer Processing Technology, Zhengzhou University, Zhengzhou 450002, China; yzfeng@zzu.edu.cn
* Correspondence: fulibi@fzu.edu.cn (L.F.); shiyq1986@fzu.edu.cn (Y.S.)
† These authors contributed equally to this work.

3. Materials and Methods

3.1. Materials and Methods

PU (65E85) was bought from Baoding Bangtai Chemical Co., Ltd. (Baoding, China). Besides, APP(II-type) powders were purchased from Shandong ChenXU New Material Co., Ltd. (Zibo, China). The precursor Ti_3AlC_2 powders (purity≥98%, 400 mesh) were purchased from 11 Technology Co., Ltd (Changchun, China). PEI(Mw=10000, 99% purity) was purchased from Aladdin Reagent Co. Ltd. (Shanghai, China). Hydrazine hydrate ($N_2H_4 \cdot H_2O$, AR) was bought from Aladdin Reagent Co. Ltd.(Shanghai, China). Concentrated hydrochloric acid (HCl, 36.5%) and lithium fluoride (LiF, 98.5% purity) were purchased from the Sinopharm Chemical Reagent Co., Ltd (Shanghai, China). GO was synthesized via Hummers' method [1]. All the above chemicals can be used without further purification.

X-ray diffraction (XRD) patterns were acquired using a DY1602/Empyrean X-ray diffractometer (Panalytical, Netherlands) with Cu Ka radiation. A Nicolet obtained Fourier transform infrared (FTIR) spectra is 50 spectrophotometer (Nicolet Instrument Company, USA) whose scanning range is 500-4000 cm^{-1}. Laser Raman spectra (LRS) were implemented in the range of 200–2000 cm^{-1} with a confocal Raman spectroscopic system (Renishaw, UK) with a 532 nm excitation wavelength. A scanning electron microscope (SEM, FEI Nova NanoSEM230, USA) was utilized for examining the morphologies of flame retardants, fractures, and char residue in TPU and its composites or composite films. These samples were sputtered with gold cladding before observation. The thermal stability of TPU and its composites was investigated with a thermal analyzer (TA Q5000, USA) ranging from 50 to 700 °C at a rate of 20 °C/min under nitrogen condition. The flame retardancy of the TPU composites was assessed using a LOI device (Model HC-2, Jiangning Analytical Instrument Co., Ltd., Nanjing, China) and TTechGBT16172-2 cone calorimetry (CCT, TESTech, Suzhou, China). The sample

dimensions for LOI and CCT are 100 × 6.7 × 3 mm³ and 100 × 100 × 3 mm³, respectively. The CFZ-2-type instrument (Jiangning Analysis Instrument Company, China) was used to conduct the vertical burning test following the ASTM D3801 standard. Additionally, each sample measuring 100 × 13 × 3 mm³ underwent five tests in the UL-94 test. The EMI SE values of TPU composite films ranging from 8 to 12 GHz (X-band) were evaluated using an N5222B vector network analyzer (Keysight Technologies, USA) at 25 °C. The sample size was 25.6 × 11.2 × 1.0 mm^3.

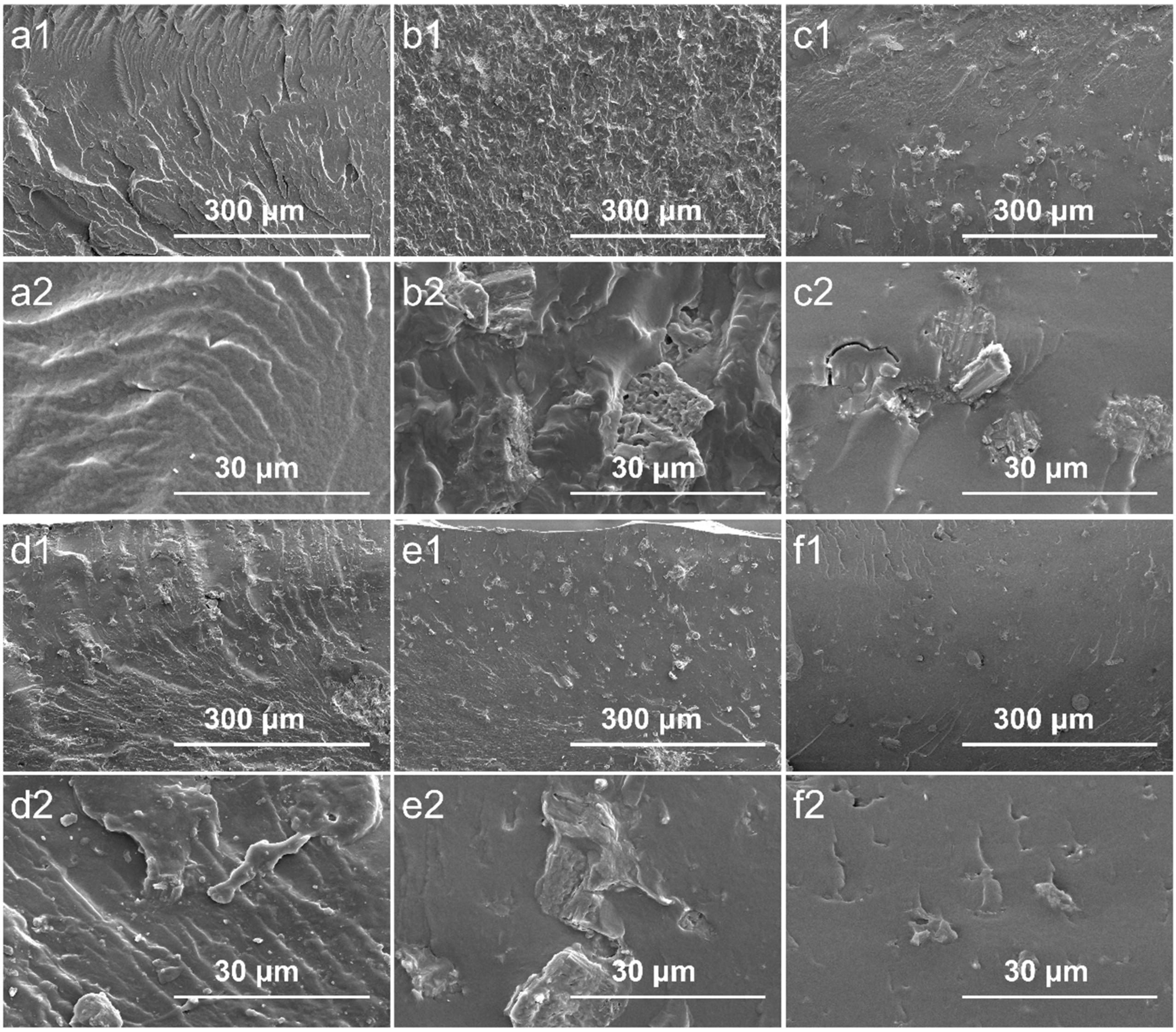

Figure S1. SEM images of the cross-sections of TPU and its composites: (a) TPU, (b) TPU/APP, (c) TPU/APP@PEI, (d) TPU/20APP@PEI@1TC, (e) TPU/10APP@PEI@1TC, and (f) TPU/5APP@PEI@1TC.

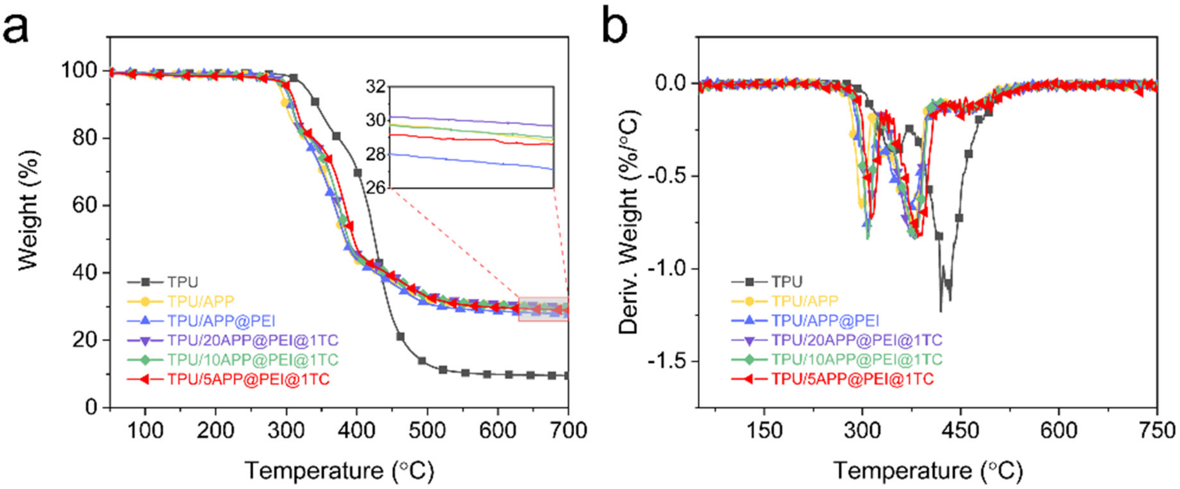

Figure S2. (a) TG and (b) DTG curves of TPU and its composites.

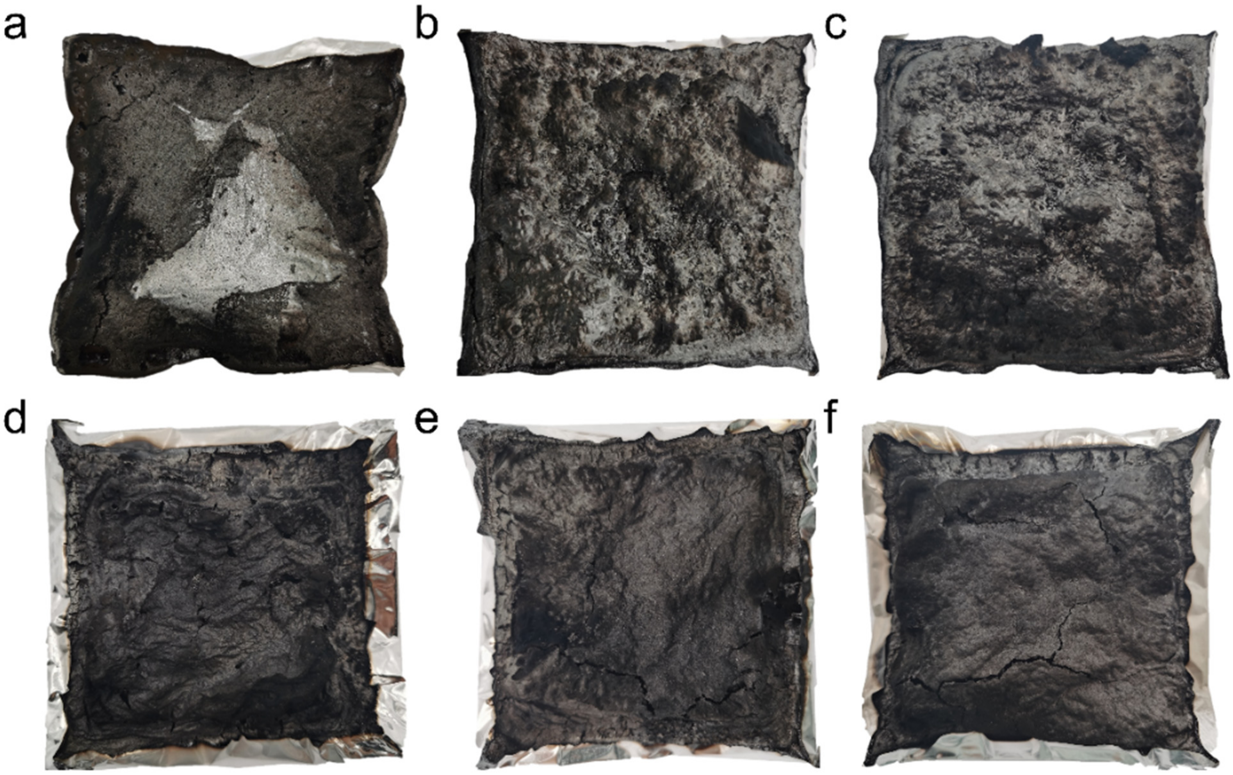

Figure S3. Digital photographs of the char residues of TPU and its composites after combustion test: (a) TPU, (b) TPU/APP, (c) TPU/APP@PEI, (d) TPU/20APP@PEI@1TC, (e) TPU/10APP@PEI@1TC, and (f) TPU/5APP@PEI@1TC.

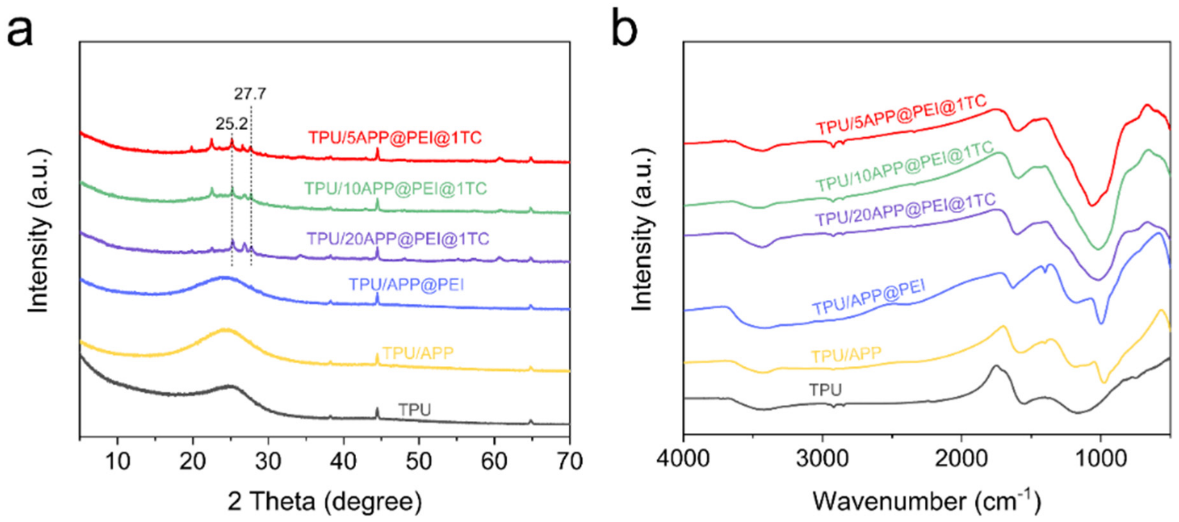

Figure S4. (a) XRD patterns and (b) FTIR spectra of char residues for TPU and its nanocomposites

Table S1. TGA test data of flame retardants under nitrogen conditions.

**Sample No.**	***T*-5 /°C**	***T*-50 /°C**	***T*max /°C**	**Residue at 700 °C/wt%**
$Ti_3C_2T_x$	475.0	/	/	94.3
APP@PEI	317.7	/	343.9	62.4
APP@PEI@$Ti_3C_2T_x$	220.1	/	314.1	76.3

Table S2. TG results of TPU and its composites under nitrogen condition.

**Sample No.**	***T*-5 /°C**	***T*-50 /°C**	***T*max /°C**			**Residue at 700 °C /wt%**
			Step1	Step2	Step3	
TPU	327.0	425.2	344.6	434.3	/	9.6
TPU/APP	290.6	383.0	298.4	374.7	454.6	29.5
TPU/APP@PEI	297.1	384.0	307.1	372.9	443.7	27.7
TPU/20APP@PEI@1TC	299.3	387.8	308.9	372.5	462.9	30.1
TPU/10APP@PEI@1TC	298.8	387.9	307.9	381.7	459.5	29.5
TPU/5APP@PEI@1TC	302.5	395.6	314.2	385.2	451.6	28.9

References

[1] H.L. Guo, M. Peng, Z.M. Zhu, L.N. Sun, Preparation of reduced graphene oxide by infrared irradiation induced photothermal reduction, Nanoscale 5(19) (2013) 9040-9048.
